# Supplementary material for: The Long-Term Evolutionary History of Gradual Reduction of CpG Dinucleotides in the SARS-CoV-2 Lineage
Source: Biology (Basel). 2021 Jan 12;10(1):52. doi: 10.3390/biology10010052 (PMC7828247; doi:10.3390/biology10010052)
Supplement: Supplementary file 1 [file biology-10-00052-s001.pdf]

Supplementary Material

The long-term evolutionary history of gradual reduction of CpG dinucleotides in the SARS-CoV-2 lineage

Sankar Subramanian[1,*]

[1] GeneCology Centre, School of Science and Engineering, University of the Sunshine Coast, Moreton Bay, Australia; ssankara@usc.edu.au

* Correspondence: ssankara@usc.edu.au

Table S1. List of Betacoronavirus genomes used in this study.

GenBank - Acc.No.	Subgenus	Host
JN874559.1	Embecovirus	Rabbit
KF294357.1	Embecovirus	Bat
KF294372.1	Embecovirus	Bat
NC_017083.1	Embecovirus	Rabbit
NC_026011.1	Embecovirus	Rat
AY597011.2	Embecovirus.	Human
KF906251.1	Embecovirus.	Camel
KY419112.1	Embecovirus.	Pig
LC061272.1	Embecovirus.	Horse
MH810163.1	Embecovirus.	Yak
NC_001846.1	Embecovirus.	Mouse
NC_003045.1	Embecovirus.	Cow
NC_006213.1	Embecovirus.	Human
NC_006577.2	Embecovirus.	Human
NC_012936.1	Embecovirus.	Rat
NC_048217.1	Embecovirus.	Rat
NC_025217.1	Hibecovirus	Bat
KX442565.1	Merbecovirus	Bat
MK907287.1	Merbecovirus	Hedgehog
MN611519.1	Merbecovirus	Bat
MN611520.1	Merbecovirus	Bat
KC545383.1	Merbecovirus.	Hedgehog
KC545386.1	Merbecovirus.	Hedgehog
KC667074.1	Merbecovirus.	Human
KC869678.4	Merbecovirus.	Bat
KJ473821.1	Merbecovirus.	Bat
KJ614529.1	Merbecovirus.	Human
MG021452.1	Merbecovirus.	Human
MG987421.1	Merbecovirus.	Human
NC_009019.1	Merbecovirus.	Bat
NC_009020.1	Merbecovirus.	Bat
NC_019843.3	Merbecovirus.	Human
NC_038294.1	Merbecovirus.	Human
NC_039207.1	Merbecovirus.	Hedgehog
EF065514.1	Nobecovirus.	Bat
EF065515.1	Nobecovirus.	Bat
EF065516.1	Nobecovirus.	Bat
HM211098.1	Nobecovirus.	Bat
HM211099.1	Nobecovirus.	Bat
HM211100.1	Nobecovirus.	Bat
HM211101.1	Nobecovirus.	Bat

MG762674.1	Nobecovirus.	Bat
NC_009021.1	Nobecovirus.	Bat
MK211374.1	Sarbecovirus	Bat
NC_014470.1	Sarbecovirus	Bat
AY304486.1	Sarbecovirus.	Civet
AY304488.1	Sarbecovirus.	Civet
AY572034.1	Sarbecovirus.	Civet
AY613950.1	Sarbecovirus.	Civet
DQ071615.1	Sarbecovirus.	Bat
DQ412042.1	Sarbecovirus.	Bat
DQ412043.1	Sarbecovirus.	Bat
JX993987.1	Sarbecovirus.	Bat
JX993988.1	Sarbecovirus.	Bat
KC881005.1	Sarbecovirus.	Bat
KC881006.1	Sarbecovirus.	Bat
KF367457.1	Sarbecovirus.	Bat
KF569996.1	Sarbecovirus.	Bat
KJ473811.1	Sarbecovirus.	Bat
KJ473812.1	Sarbecovirus.	Bat
KJ473813.1	Sarbecovirus.	Bat
KJ473814.1	Sarbecovirus.	Bat
KJ473815.1	Sarbecovirus.	Bat
KJ473816.1	Sarbecovirus.	Bat
KP886808.1	Sarbecovirus.	Bat
KP886809.1	Sarbecovirus.	Bat
KY352407.1	Sarbecovirus.	Bat
MG772933.1	Sarbecovirus.	Bat
MG772934.1	Sarbecovirus.	Bat
MN996532.1	Sarbecovirus.	Bat
MT040333.1	Sarbecovirus.	Pangolin
MT040334.1	Sarbecovirus.	Pangolin
MT040335.1	Sarbecovirus.	Pangolin
MT040336.1	Sarbecovirus.	Pangolin
MT072864.1	Sarbecovirus.	Pangolin
MT121216.1	Sarbecovirus.	Pangolin
MT782114.1	Sarbecovirus.	Bat
NC_004718.3	Sarbecovirus.	Human
NC_045512.2 (SARS-Cov-2)	Sarbecovirus.	Human, mink, tiger, dog and cat

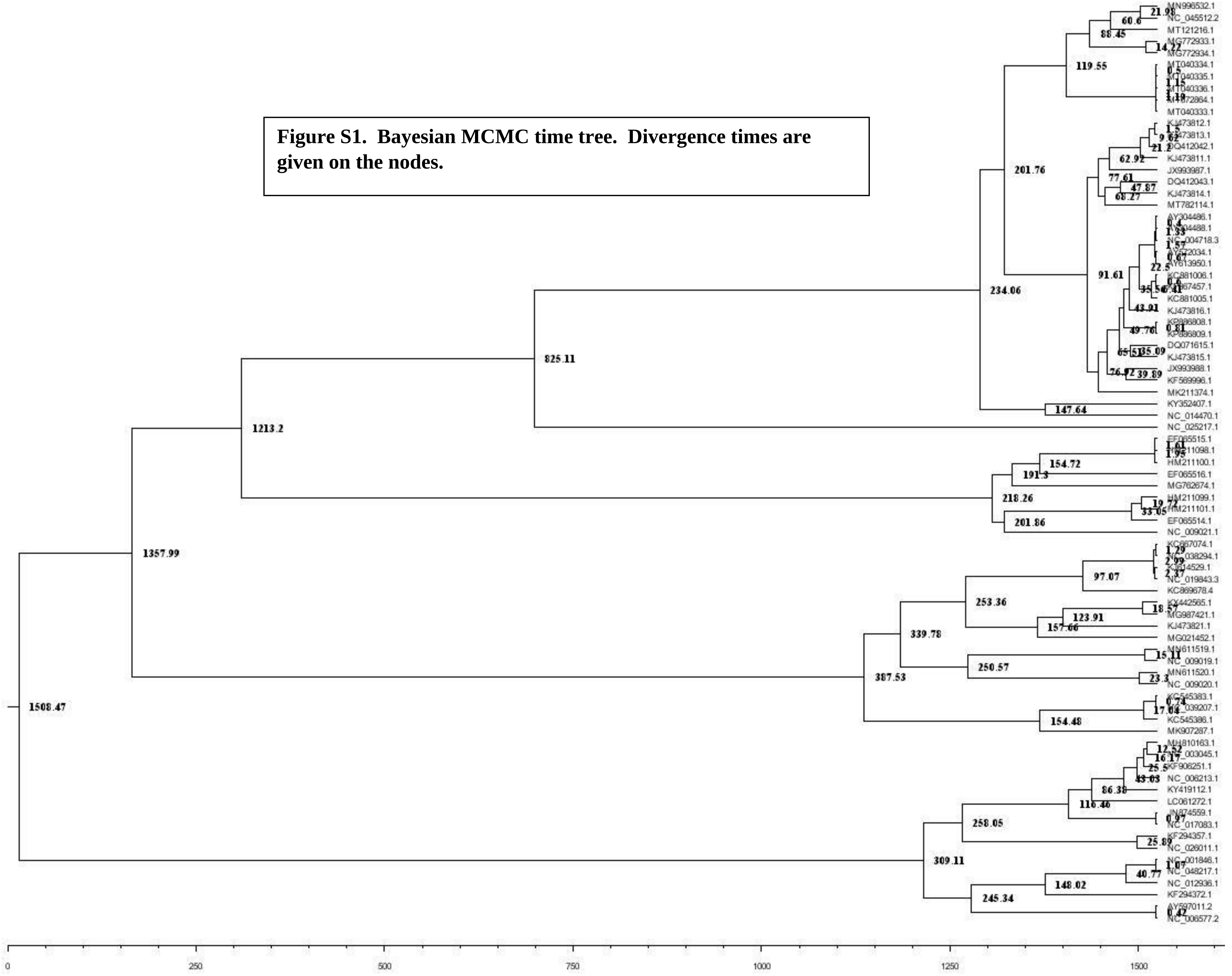

Figure S1. Bayesian MCMC time tree. Divergence times are given on the nodes.
